# Mammalian Cells Undergo Endoreduplication in Response to Lactic Acidosis

**DOI:** 10.1038/s41598-018-20186-7

**Published:** 2018-02-13

**Authors:** Zhihao Tan, De Zhi Valerie Chu, Yong Jie Andrew Chan, Yi Ena Lu, Giulia Rancati

**Affiliations:** 1 Institute of Medical Biology, Agency for Science, Technology and Research (A*STAR), Singapore, Singapore; 2 Present Address: Genome Institute of Singapore, Agency for Science, Technology and Research (A*STAR), Singapore, Singapore

## Abstract

Polyploidization, a common event during the evolution of different tumours, has been proposed to confer selective advantages to tumour cells by increasing the occurrence of mutations promoting cancer progression and by conferring chemotherapy resistance. While conditions leading to polyploidy in cancer cells have been described, a general mechanism explaining the incidence of this karyotypic change in tumours is still missing. In this study, we tested whether a widespread tumour microenvironmental condition, low pH, could induce polyploidization in mammalian cells. We found that an acidic microenvironment, in the range of what is commonly observed in tumours, together with the addition of lactic acid, induced polyploidization in transformed and non-transformed human cell lines *in vitro*. In addition, we provide evidence that polyploidization was mainly driven through the process of endoreduplication, i.e. the complete skipping of mitosis in-between two S-phases. These findings suggest that acidic environments, which characterize solid tumours, are a plausible path leading to polyploidization of cancer cells.

## Introduction

Tetraploidization is a frequent event during cancer evolution. Evidence of widespread presence of polyploid cells comes from the observations that many tumours display a bimodal distribution of chromosome numbers, with the first peak centred on a near-diploid karyotype and the second between that of a triploid and tetraploid genome^1–3^. Even if many cancer cells are chromosomally unstable and tend to gain and lose chromosomes at high frequency^4^, whole-genome duplication is the most likely explanation for the presence of cancer cells with a ploidy >3 N. Moreover, flow cytometry, cytogenetic and histological analyses from patients or murine models affected by a variety of solid tumours provided experimental evidence that tetraploidization is commonly present in tumour samples even at early stages of its progression^5–10^. Tetraploidization has been positively correlated with cancer evolution and proposed to promote its progression at different stages. Indeed, p53-null tetraploid but not diploid mouse mammary epithelial cells were shown to induce malignant mammary epithelial transformation when transplanted subcutaneously into nude mice^11^, suggesting that tetraploid cells could help kick-start the transformation process. Moreover, tetraploidization has been shown to confer increased drug resistance to certain classes of cytotoxic drugs or chemotherapy compounds currently used in the clinic^12,13^ (Tan, Chan and Rancati, *unpublished observations*), suggesting that they might play an important role also during emergence of drug resistance. Furthermore, it has been shown that genome doubling can lead to the formation of aneuploid cells due to the presence of extra centrosomes and multipolar spindle intermediates^14,15^. Since aneuploid cells have been associated with tumour progression^4^, this observation suggests that polyploidization could be an intermediate step in the progression of normal tissues into highly aneuploid cancers displaying chromosomal instability. This hypothesis has been supported by data from colorectal^16^, breast^5^, cervical^6^ and bladder cancers^8^.

There are currently four known paths leading to tetraploid cells from diploid precursors^1^: cell-to-cell fusion, cytokinesis failure, mitotic slippage and endoreduplication. While many of these processes occur in normal tissues and are part of the development of specialized cells, some of them have been shown or hypothesized to occur during tumour development. For instance, cell-to-cell fusion, a process in which cell membranes of adjacent cells fuse together, has been reported to increase transformation *in vitro*^17^ and occur after viral infection of oncogenic viruses such as the human papilloma virus^18^. Cytokinesis failure after complete nuclear segregation has been shown to occur in response to common events in cancer progression, such as overexpression of mitotic regulators or presence of lagging chromosomes^1^. Mitotic slippage was reported in conditions that mimic chemotherapy treatment such as after prolonged incubation with antimitotic drugs^19^. Lastly, endoreduplication, a process in which cells completely skip mitosis and enter into the subsequent G1 phase, was shown to occur during persistent telomere damage and dysfunction^20^, which are common steps during tumour evolution.

Despite the above links between polyploidization and transformation, a general mechanism explaining how this karyotypic change occurs during tumour progression is still missing. Since cancer cells display a large intra- and inter-tumour heterogeneity^21^, we searched for paths leading to tetraploidization in cancer features that are common to different tumours. In single-cell eukaryotes and plants, it has been recently shown that stressful environments could induce karyotypic changes. For example, heat shock in *S. cerevisiae* and drug stress in *C. albicans* was shown to induce aneuploidy^22,23^; insufficient light, cold stress, drought or exposure to pathogens can induce plants to polyploidize various tissues^24^. A near universal stress found in solid tumours is the presence of an acidic microenvironment^25^. While non-transformed adult cells have an extracellular pH (pH_e_) of ~7.4, cancer cells have a lower average pH_e_ of ~6.7–7.1^25^, with pH_e_ as low as 5.8 being reported^26^. This acidic environment is primarily generated by a combination of two effects. On one hand, cancer cells display an altered metabolism^27^ and export large amounts of lactate and protons, thereby acidifying the extracellular environment. On the other hand, poor vascularization and blood perfusion of the tumour mass leads to reduced gas exchange and accumulation of H^+^ ions in the extracellular environment. The combination of these two factors has been hypothesized to be at the basis of the observed reduced pH_e_ in solid tumours^27^.

We therefore tested whether acidic microenvironments could trigger polyploidization as a stress response in mammalian cells. In this paper, we report that lactic acidosis alone induced tetraploidization in transformed and non-transformed human cell lines *in vitro*. Using the Fluorescent Ubiquitination-based Cell Cycle Indicator (FUCCI) system, we provide evidence that tetraploidization mostly occurs through the process of endoreduplication. Since acidosis is a hallmark of solid tumours, these observations suggest that acidic microenvironments are a potential common route to polyploidization of cancer cells in different solid tumours.

## Results

### Optimised conditions for acidic tissue culture media

**Figure 1:**
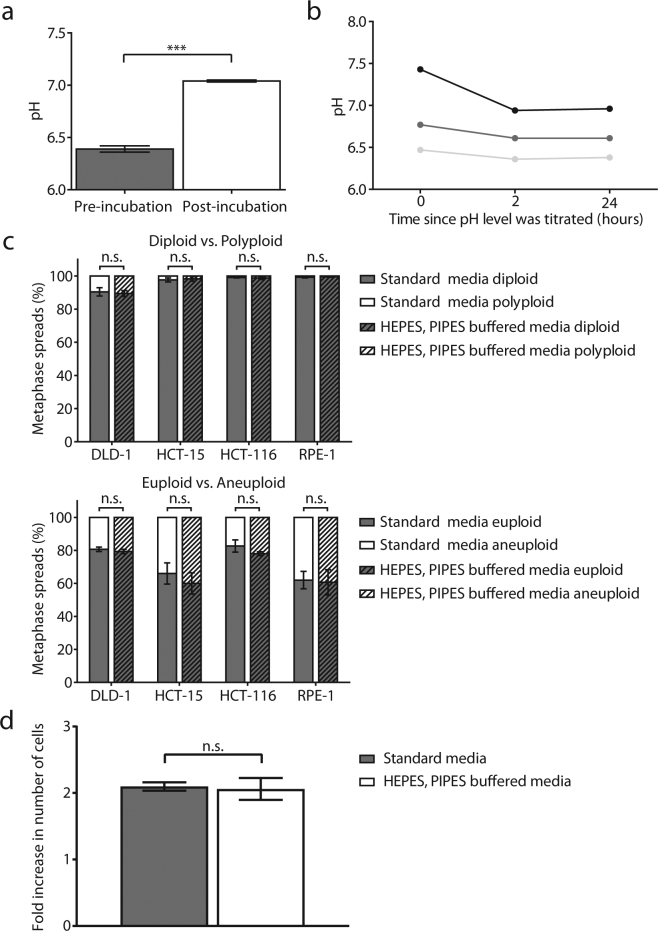
A combination of 10 mM HEPES and 10 mM PIPES is an optimised buffer system for acidic growth conditions. (**a**) pH levels of McCoy’s 5A media containing sodium bicarbonate before (right after pH titration, left column) and at the end (right column) of a 8-hour incubation in a 5% CO_2_ incubator. N = 3 independent replicates, unpaired t-test, p-values: *** < 0.001. (**b**) pH levels of 10 mM HEPES, 10 mM PIPES buffered media. 3 different starting pH levels were tested, and media was incubated in a 5% CO_2_ incubator for the indicated time period. The slight pH decrease of the media upon 5% CO_2_ incubation could be ascribed to CO_2_ solubilization and consequent carbonic acid formation (**c**) Relative frequency of diploid/polyploid (top panel) and euploid/aneuploid (bottom panel) cells after 1 day of incubation in standard or HEPES/PIPES buffer media in the indicated cell lines. N = 3 biological replicates, >100 cells per replicate were analysed for the top panel; 50 cells per replicate were analysed for the bottom panel; statistical analysis: unpaired t-test. (**d**) Fold increase in number of DLD-1 cells incubated with either standard or HEPES/PIPES buffer media over 24 hours. 5 × 10^5^ cells were seeded and grown for one day before exposure with the indicated media. Standard media refers to media with sodium bicarbonate buffer system. N = 3 biological replicates, SEM are depicted as error bars. Unpaired t-test, n.s.: not significant.

Current pre-made liquid cell-culture media contain a buffer system that maintains a stable pH between ~7.0 and ~7.4 in a 5% CO_2_ incubator. The buffer system is based on the equilibrium between an optimised amount of sodium bicarbonate and atmospheric CO_2_. In presence of increased H^+^ ions and acidic conditions, the pH is readily stabilised back at ~7.0–7.4 (Fig. 1a) by increasing concentration of HCO_3_^−^ ions in the media. Therefore media using this buffer system are not suitable for growth of mammalian cells at pH ranges different from ~7.0–7.4. We thus had to replace sodium bicarbonate with a buffer system that can stably maintain acidic pH levels.

**Figure 2:**
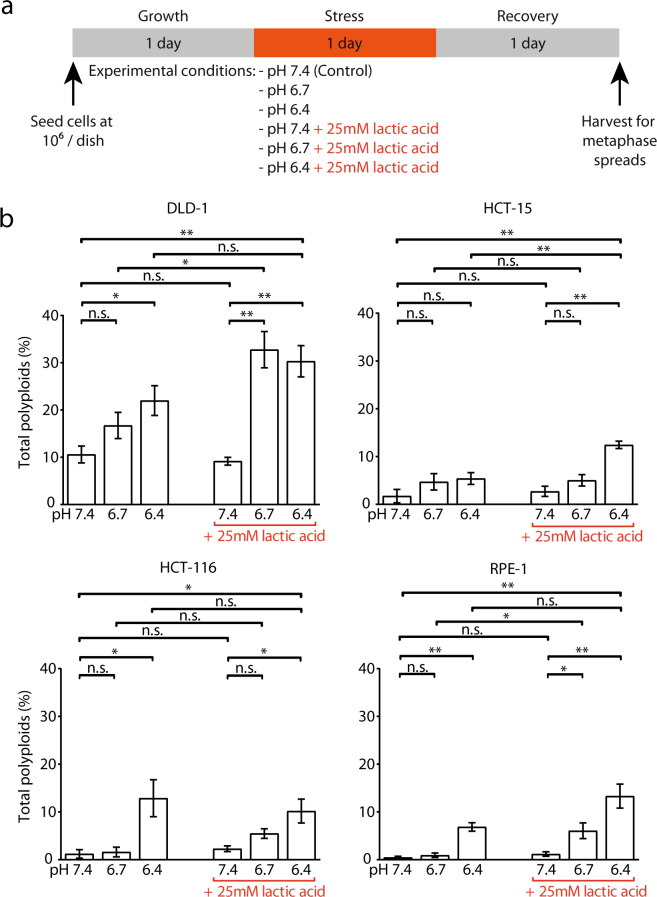
Acidic environments increase the proportion of polyploid cells. (**a**) Diagram illustrating the different treatment regimens. 10^6^ cells were seeded and grown for one day before a 24-hour exposure with the indicated media. 24-hour after the release from stress, cells were harvested for metaphase spread preparation. (**b**) Percentage of polyploid cells in the tested cell lines after exposure to indicated regimens. N ≥ 3 biological replicates, > 100 cells per replicate were analysed; SEM are depicted as error bars. Unpaired t-test, p-values: * < 0.05, ** < 0.01, *** < 0.001, n.s.: not significant.

**Figure 3:**
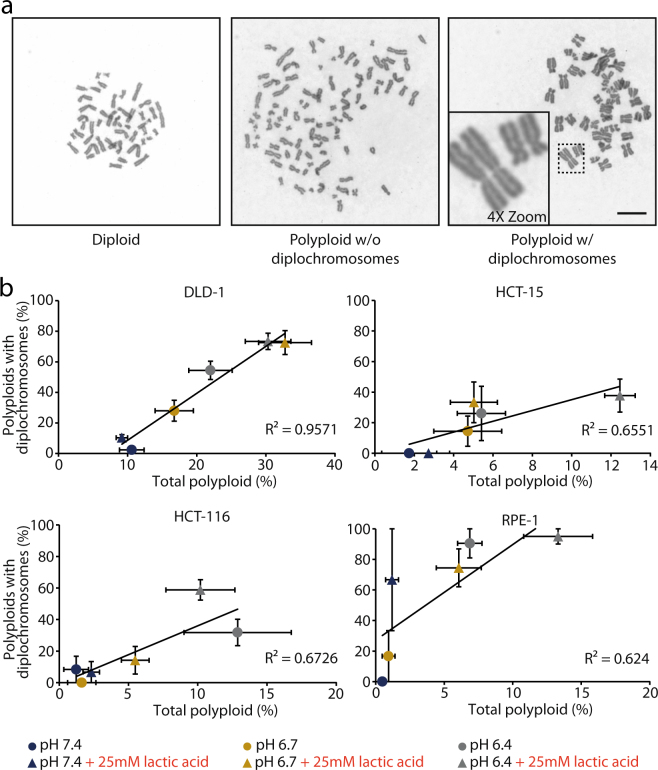
Acidic-triggered polyploid cells carried diplochromosomes. (**a**) Representative metaphase spreads showing a diploid cell (left) and a polyploid cell without (middle) or with (right) diplochromosomes. The inset in the bottom of the right panel shows magnification of two couples of diplochromosomes. All images were acquired using the same magnification; scale bar: 10 µm. (**b**) Scatter plots displaying the percentage of cells with diplochromosomes among all polyploid cells versus the percentage of polyploid cells among all analysed cells, following exposure to the indicated regimens. The large error bars in some data points are due to the low total number of polyploid cells in that sample. R^2^ is reported in each panel. N ≥ 3 biological replicates, >100 cells per replicate were analysed; error bars represent SEM.

HEPES (4-(2-hydroxyethyl)-1-piperazineethanesulfonic acid) and PIPES (1,4- piperazinediethanesulfonic acid) are commonly-used, well-tolerated buffering agents with a buffering capacity in the range of pH ~6.8–8.2 and pH ~6.1–7.5, respectively. We thereby selected a buffer system that relies on a combination of these two chemicals at a concentration not known to be toxic for mammalian cells (see Material and Methods). This buffer system stably maintained the pH of the media (Fig. 1b, especially at lower pH) and had no noticeable effects on cell morphology (Supplementary Fig. S1), on karyotypic stability (Fig. 1c) or on cellular growth over a 24-hour incubation (Fig. 1d) when tested at pH 7.4. We therefore concluded that this buffer system was non-toxic, maintained a fairly stable pH and was suitable for use throughout this study. Moreover, to confirm the generality of our findings, all experiments presented in Figs 2 and 3 have also been repeated with media that utilised a 15 mM Bis-Tris (2,2-Bis(hydroxymethyl)-2,2′,2″-nitrilotriethanol) buffer system with qualitatively similar results (Supplementary Fig. S2, Supplementary Fig. S3).

### Lactic acidosis induced polyploidization in transformed and non-transformed cell lines

To test if karyotypic changes could be triggered by acidic environments, cells were exposed to acidic (pH 6.4 or 6.7) or control (pH 7.4) media for 24 hours. Subsequently, cells were harvested for chromosome counting 24 hours after they were returned to standard culturing conditions. Since acidic tumour environments can contain up to 40 mM of lactic acid^28^, the same regimen was applied in presence or absence of 25 mM of lactic acid (Fig. 2a). It should be noted that the lactic acid was added before titration of the media, thus the pH of the experimental media is as stated. This stress regimen was tested on multiple chromosomally stable human cell lines (such as DLD-1, HCT-15 or HCT-116 cancer cells, or the non-transformed retinal epithelial cells RPE-1), all of which were either diploid or pseudodiploid.

Chromosome counting on metaphase spreads showed that exposure to different acidic environments in presence or absence of lactic acid increased the proportion of polyploid cells (defined as cells with ≥66 chromosomes, see Materials and Methods) in the majority of tested cell lines (Fig. 2b). Though this observation is in contrast with a previous report showing that acidosis *per se* does not trigger polyploidization^29^, we note that the cell culturing conditions used in our study are different and have been optimised for pH stabilization of the media. While addition of lactic acid by itself did not change the cellular karyotype (Fig. 2b, compare pH 7.4 lane vs. pH 7.4 + 25 mM lactic acid lane), it often led to an increased amount of polyploid cells when combined with lower pH levels (Fig. 2b, see DLD-1, HCT-15 and RPE-1). This observation suggests that lactate molecules in the tumour microenvironment might work as an active signal to trigger polyploidization more than just contributing to this karyotypic change by lowering the pH. In contrast, the application of this stress regimen in presence or absence of lactic acid did not alter the proportion of aneuploid cells (defined as cells with a non-modal chromosome count of <66 chromosomes, Supplementary Fig. S4), suggesting that polyploidization is not the result of an increased chromosome instability.

### Polyploidization arose from endoreduplication events

Endoreduplication is a process by which cells undergo two rounds of DNA replication without entering mitosis and dissolving centromeric cohesion^30,31^. Following endoreduplication, metaphase spreads contain diplochromosomes, which are chromosomal structures characterised by four sister chromatids held together (Fig. 3a). Metaphase spread analysis after acid treatment showed that increasing percentages of polyploidization were accompanied by an increase of polyploid cells carrying diplochromosomes (Fig. 3b), suggesting that polyploidization was mostly occurring through endoreduplication. To confirm this, we performed live-cell imaging on cell cycle progression of cells exposed to lactic acidosis using FUCCI. The FUCCI system relies on fragments of specific cell cycle proteins tagged with different fluorophores and therefore cells expressing this construct show different fluorescence colours at different stages of cell cycle progression^32,33^. Specifically for the implemented system that we utilised in this study, G1 cells appear red as they express mCherry-hCdt1 (hCdt1 amino acid residues 30/120), G2/M cells appeared green as they express mAG-hGeminin (hGeminin amino acid residues 1/110), while S phase cells are yellow as they express a combination of the two proteins. Upon endoreduplication, cells will cycle from G2 to G1 (from green to red fluorescence) without physically rounding up or separating (indicating that no mitosis occurred).

**Figure 4:**
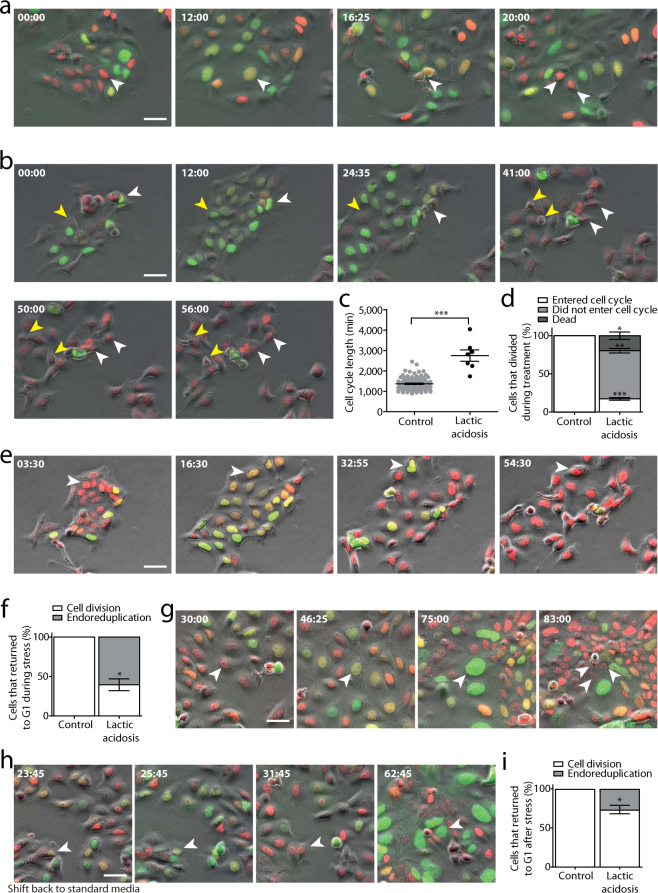
Acidic-driven polyploidization occurred through endoreduplication. (**a**) Highlighted cell completes cell division in control media. (**b**) Cell cycle progression in lactic acidosis media. White arrowheads: cell with a lengthened cell cycle followed by G1 arrest of at least ~32 hours (from 24:35 to 56:00). Yellow arrowheads: daughter cells at 41:00 with a lengthened cell cycle followed by cell death ~15 hours after cell division (56:00). (**c**) Cell cycle length quantification (from G1 to cytokinesis) in control or lactic acidosis media. Control N = 86, stress N = 7. (**d**) Quantification of cell fate following cell division in control or lactic acidosis media. Cells were classified as not entering the cell cycle if they remained in G1 phase for at least 24 hours after cell division. N = 2 biological replicates, ≥45 cells were analysed per condition per replicate. (**e**) The indicated cell undergo endoreduplication in presence of lactic acidosis stress. (**f**) Frequency of cells undergoing cell division or endoreduplication in control or lactic acidosis media. N = 2 biological replicates, ≥50 cells were analysed per condition per replicate. (**g**) Following release into standard media (23:50), the indicated endoreduplicated cell re-entered the cell cycle (46:25) and underwent multipolar mitosis (83:00). (**h**) Arrowheads: S/G2-phase cell at the time of media replacement from lactic acidosis to control (23:45) that underwent endoreduplication resetting to G1 (31:45) and progressed through a novel cell cycle (G2-phase at 62:45). (**i**) Quantification of S- and G2-phase cells undergoing cell division or endoreduplication in their first cell cycle upon release from lactic acidosis. For lactic acidosis, cells were scored after the release from lactic acidosis (24:00). For control, cells unexposed to lactic acidosis media were scored from 24:00 after the beginning of the imaging experiment. N = 2 biological replicates, ≥25 cells were analysed per condition per replicate. Unpaired t-test, p-values: * < 0.05, ** < 0.01, *** < 0.001; error bars represent SEM. Time displayed in hh:mm, with 00:00 corresponding to beginning of video. Scale bar: 50 µm. Full videos are provided as Supplementary Videos S1–5.

In control media, FUCCI-tagged DLD-1 cells displayed a typical cell cycle progression. Initially, red G1-phase cells progressed to yellow S-phase and then to green G2-phase cells before undergoing mitotic rounding up and cell division (Fig. 4a and Supplementary Video S1). The duration of the cell cycle was qualitatively comparable with untagged DLD-1 cells (data not shown). When FUCCI-tagged DLD-1 cells were imaged during continuous exposure to lactic acidosis stress, we noticed several changes. Firstly, there was a delay in the cell cycle progression; for example the cell marked with a yellow arrowhead in Fig. 4b divided at 41:00 despite being in G2/M for at least 30 hours. Secondly, upon cell division, a large proportion of cells either arrested in G1 or underwent cell death; for example the cells indicated with yellow arrowheads undergo cell death at 56:00 (Fig. 4b–d and Supplementary Video S2). Interestingly, among the cells that re-entered another cell cycle in presence of lactic acidosis, the majority of them underwent endoreduplication and progressed from G2 to G1 without undergoing mitosis (Fig. 4e,f and Supplementary Video S3). We then asked whether cells that have undergone endoreduplication re-enter a new cell cycle and divide when the conditions are shifted back to standard (control) media. To this end, cells that had been exposed to lactic acid for 23 hours and 50 minutes were shifted to standard conditions until the end of the imaging experiment. Upon release from the acidic stress, the majority of cells that underwent endoreduplication restarted their cell cycle (Fig. 4g and Supplementary Video S4). For example, the indicated cell in Fig. 4g entered G2/M at 46:25 roughly 20 hours after the shift back to standard media and underwent tripolar mitosis at 83:00. These observations suggested that lactic acidosis induces polyploidization mostly though endoreduplication. As a result, polyploid cells arising from endoreduplication could progress through mitosis upon stress release and could become chromosomally unstable by undergoing aberrant mitosis. We also noted that, upon release from stress, ~25% of S- or G2-phase cells that were previously exposed to acidic environments underwent endoreduplication (Fig. 4h-i and Supplementary Video S5), suggesting that exposure to acidic environments could have long-lasting effects on the genome instability of exposed cells.

## Discussion

Though polyploidy has been positively correlated with early as well as late stages of tumour evolution^11–13^ and contributes to the rampant aneuploid population in cancers^14,15^, we still miss a general mechanism describing how cancer cells increase their ploidy. Here, we showed that *in vitro* exposure of transformed or non-transformed human cells to fluctuating acidic environments was sufficient to trigger genome doubling through the process of endoreduplication. We also demonstrated that some of the acidic-triggered polyploid cells underwent multipolar mitosis, likely causing chromosome instability. Given the fact that acidosis is a general feature of solid tumours, these observations suggest that *in vivo* acidic tumour microenvironments are a potential common trigger towards polyploidization and aneuploidization. Moreover, tetraploidization related to cancer cells has been shown to result from either viral-mediated infection^17^, presence of lagging chromosome at the metaphase plate^34^, persistent DNA damage due to telomere attrition^20^ and dysregulation of mitotic proteins (i.e.: Mad2, Aurora A) or tumour suppressors (i.e.: Adenomatous Polyposis Coli)^1^. The acidic-driven tetraploidization described in this manuscript adds to these observations and is conceptually different from what previously reported. Indeed, the acidic-driven tetraploidization does not rely on any intrinsic cellular factors, such as mutations or chromosome alignment issues. As such, exposure to acidic environments is sufficient to drive tetraploidization regardless of the genetic background or chromosome instability status of the exposed cells. As tumours form, acidic microenvironments are generated as a result of an increased lactate secretion from anaerobic glycolysis and decreased blood perfusion. It is known that acidification of the microenvironment contributes to tumour evolution by promoting angiogenesis and degradation of the extracellular matrix^27^. The findings in this study provide an additional connection between acidosis and tumour evolution. Acidic environments could trigger polyploidization and subsequent generation of aneuploid cells, which in turn have both been shown to promote tumour evolution and drug resistance^4,13^. On possible implications for chemotherapy, a few recent reports indicated that an increased pH in tumour masses inhibit or delay the formation of metastasis^35–37^. Since centrosome amplification, a hallmark of polyploid cells, has been shown to induce cellular invasion^38^, we speculate that treatments aiming at reducing the acidification of the tumour microenvironment might curb metastasis by decreasing the incidence of polyploid cells in the tumour.

While p53 was shown to restrain the growth of polyploid cells^39^, we were able to detect endoreduplication-induced tetraploid cells using metaphase spreads in a non-transformed RPE-1 cell line with intact p53 signalling cascade. This observation suggests that at least some polyploid cells formed through the process of endoreduplication might proliferate despite a wild-type p53 signalling cascade. Endoreduplication occurs during the development of specialized cells such as trophoblast giant cells in mammals^40^. Therefore we speculate that endoreduplication-derived polyploid cells might be insensitive to the p53-mediated G1 arrest as endoreduplication occurs normally during development. Future studies will be required to follow up the long-term fate of polyploid cells derived from endoreduplication events in presence or absence of an active p53 signalling cascade. Confirmation of the ability of endoreduplication-derived polyploid cells to proliferate despite an intact p53 signalling cascade could have important implications in our understanding of what triggers p53 activation in polyploid cells formed by other mechanisms. Moreover, if polyploidization in cancer cells is mostly driven by endoreduplication, our observations would imply that the p53 status of tumour cells does not affect the proliferation capacity of tetraploid cells. This would extend the generality of our finding and make them applicable to virtually all solid tumours.

Lastly, the finding that cells can endoreduplicate even after the acidic stress is removed suggests that endoreduplication could be boosted by fluctuating acidic environments, which are typical of tumours. Moreover, factors contributing to the observed tetraploidization might have long-lasting effects on cell cycle progression. The time needed for the transcriptional activation of effector genes or the accumulation or degradation of a molecular signal to a certain threshold might be possible explanations for the temporal lag seen between the acidic stimulus and tetraploidization. The process of endoreduplication has been well studied in normal development, e.g. the cyclin E-Cdk2 cycles of trophoblast giant cells^41^. It would be interesting to investigate the possibility that the same pathways are hijacked by an acidic microenvironment. Alternatively, it has also been seen that endoreduplication can be induced through drugs (e.g. DNA hypomethylating agent 5-azaC^42^, phosphatase 2A inhibitor okadaic acid^43^, arsenite^44^) and disruption of the cell cycle^45–47^. Further investigation in these areas could provide hints to the molecular mechanism that underlies this phenomenon of acidosis-induced endoreduplication.

## Materials and Methods

### Cell lines and cell culture conditions

Colorectal carcinoma cell lines HCT-116 (CCL-247), HCT-15 (CCL-225), DLD-1 (CCL-221) and hTERT RPE-1 (CRL-4000) were obtained from American Type Culture Collection and were passaged in standard conditions and recommended standard media (DLD-1 and HCT-15: RPMI1640; HCT-116: McCoy’s 5A; RPE-1: DMEM/F-12 mix) unless otherwise specified. Experimental media was prepared from powdered media (RPMI1640, Gibco 31800; McCoy’s 5A, Sigma M4892; DMEM/F-12, Thermo 12500), with the addition of 10 mM HEPES (Sigma) and 10 mM PIPES (Sigma) or 15 mM Bis-Tris (Sigma), 10% FBS (Gibco), and additional supplements to match the formula of the pre-made liquid media. 25 mM L-(+)-lactic acid (Sigma) was optionally added.

### Metaphase spreads

KaryoMAX Colcemid (Gibco) was used for metaphase spread preparation. Following incubation with Colcemid for 2 to 4 hours, cells were harvested by trypsinization and washed once in PBS. Cells were gently resuspended in ~100 μl of PBS, and 75 mM potassium chloride (Kanto) was slowly added up to a volume of 10 ml. The samples were then incubated for 10 min in a 37 °C water bath. Following that, 1 ml of Carnoy solution (3:1 Methanol:Acetic acid) was added to each sample, and samples were centrifuged for 10 min at 1,000 rpm. Supernatant was discarded and cells were gently resuspended in a small amount of remaining supernatant. 10 ml of Carnoy solution was slowly added to the samples and cells were fixed for at least 1 hour at room temperature (RT). Samples were centrifuged for 10 min at 1,000 rpm, supernatant was discarded, and cells were fixed again with 10 ml of fresh Carnoy for at least 1 hour at RT. Samples were then centrifuged and pellets were resuspended in a suitable volume (~0.5–1.5 ml) of fixative. 20 μl of this suspension was drawn up and dropped from a height (10–30 cm) onto glass slides and allowed to air-dry. DNA was then stained with Giemsa (Gibco) and imaged. To obtain polyploid cell proportions, chromosome spreads were quantified as polyploid if the chromosome count was ≥66 or as diploid if the chromosome count was <66. To obtain aneuploid cell proportions, only chromosome spreads of diploid cells were quantified and cells were classified as aneuploid if the chromosome count was not the modal number of the specific cell line or as euploid if the chromosome count matched the modal number. DLD-1, HCT-15, RPE-1 display a modal chromosome count of 46, whereas HCT-116 of 45.

### FUCCI

FUCCI plasmids were provided by the Miyawaki lab and characterised previously^48^. Each FUCCI plasmid (mCherry-hCdt(30/120)/pCSII-EF-MCS, mAG-hGeminin(1/110)/pCSII-EF-MCS) was co-transfected into 293T cells with suitable packaging vectors for viral expression. Harvested viruses were sequentially transduced into DLD-1 cells. FACS (Fluorescence-activated cell sorting) was used to select cells expressing fluorophore of interest at each stage.

### Imaging

Metaphase spreads were visualised with an Imager.Z2 (Zeiss) and imaged with a CoolCube 1 (Metasystems) and analysed using ImageJ. Live-cell imaging was performed on an IX81 (Olympus) fitted with a CO_2_ Module S (Pecon) and a JCS controller (Shinko) for CO_2_ and temperature control respectively, and imaged with a CoolSnap HQ (Photometrics). Live-cell imaging videos were recorded up to 120 hours. Images were acquired every 5 min. Exposures were as follow: Phase contrast 30 ms, GFP 100 ms and RFP 100 ms. 20× magnification lenses were used for the acquisition of the live-imaging videos.

## Electronic supplementary material

Supplementary information

Supplementary Video S1

Supplementary Video S2

Supplementary Video S3

Supplementary Video S4

Supplementary Video S5
