# Supplementary material for: Mammalian Cells Undergo Endoreduplication in Response to Lactic Acidosis
Source: Sci Rep. 2018 Feb 13;8:2890. doi: 10.1038/s41598-018-20186-7 (PMC5811548; doi:10.1038/s41598-018-20186-7)

Supplementary information for:

Mammalian Cells Undergo Endoreduplication in Response to Lactic Acidosis

Zhihao Tan^1,2^, De Zhi Valerie Chu^1^, Yong Jie Andrew Chan^1^, Yi Ena Lu^1^, Giulia Rancati^1^,*

^1^ Institute of Medical Biology, Agency for Science, Technology and Research, Singapore

^2^ Current Address: Genome Institute of Singapore, Agency for Science, Technology and Research, Singapore

Zhihao Tan and De Zhi Valerie Chu contributed equally to this work

* Corresponding author:

Giulia Rancati

Phone: +65 6407 0805

E-mail: [giulia.rancati@imb.a-star.edu.sg](mailto:giulia.rancati@imb.a-star.edu.sg)

**Supplementary Figure S1 - Cell morphology of cell lines used in the present study in either 10mM HEPES and 10mM PIPES buffered or standard media.** Images of DLD-1, HCT-15, HCT-116 and RPE-1 cell lines after 1 day incubation in either standard media (pre-made liquid media, see **Materials and Methods**) or 10mM HEPES and 10mM PIPES buffered media. All images were acquired using the same magnification; scale bar: 0.2mm.

**Supplementary Figure S2 – Acidic media buffered using 15mM Bis-Tris increased the proportion of polyploid cells in the majority of tested cell lines.** Stress regimens are similar to Figure 2a but media was buffered using 15mM Bis-Tris. Standard media refers to pre-made tissue culture media buffered with sodium bicarbonate. N ≥ 3 biological replicates, >100 cells per replicate were analysed; SEM are depicted as error bars. Unpaired t-test, p-values: * < 0.05, ** < 0.01, *** < 0.001.

**Supplementary Figure S3 – Lactic acidic stresses induced the formation of diplochromosomes.** Percentage of polyploid cells displaying diplochromosomes after acidic stress regimens over the total polyploid cells found as described in Supplementary Figure S2. In the bottom right panel (RPE-1) the data points for “standard media”, “pH 7.2” and “pH 7.2 + 25mM lactic” acid overlap on the x axis. N ≥ 3 biological replicates, >100 cells per replicate were analysed; SEM are depicted as error bars. Unpaired t-test, p-values: * < 0.05, ** < 0.01, *** < 0.001.

**Supplementary Figure S4 – Acidic environments did not trigger aneuploidization in the tested cell lines.** Graphs refer to the percentage of euploid and aneuploid cells for the indicated cell lines after being exposed to stress regimens described in Figure 2a. N ≥ 3 biological replicates, 50 cells per replicate were analysed; SEM are depicted as error bars. Unpaired t-test, p-values: ** < 0.01.

**Supplementary Video S1 – Cell cycle progression of FUCCI-transfected DLD-1 cells in control media at pH 7.4.** White arrowhead highlight a G1 cell progressing through G2 and cell division during the duration of the video. Snapshots of this video are reported in Figure 4a.

**Supplementary Video S2 – Cell cycle progression of FUCCI-transfected DLD-1 cells in lactic acid media at pH 6.4.** The white arrowhead highlights a G1 cell dividing into two daughter cells which arrest in G1 for the entire duration of the video. The green arrowhead highlights a G1 cell dividing into two daughter cells which arrest in G1 and subsequently round up and die before the end of the video. Snapshots of this video are reported in Figure 4b.

**Supplementary Video S3 – Endoreduplication in response to continuous lactic acidosis stress.** The white arrowhead highlights a cell undergoing endoreduplication and going from G2 (green) to G1 (red) without an intervening mitosis. Snapshots of this video are reported in Figure 4e.

**Supplementary Video S4 – Multipolar mitosis following an endoreduplication event.** The arrowhead indicates a cell undergoing endoreduplication during the lactic acid exposure (before 23:50). Upon restoration of standard conditions at 23:50, the indicated endoreduplicated cell re-entered the cell cycle and underwent multipolar mitosis. Selected snapshots of this video are reported in Figure 4g.

**Supplementary Video S5 – Endoreduplication after restoration of standard culturing media.** After restoration of standard culturing conditions at 23:45, the indicated G1 cell underwent endoreduplication at 31:45. Snapshots of this video are reported in Figure 4h.


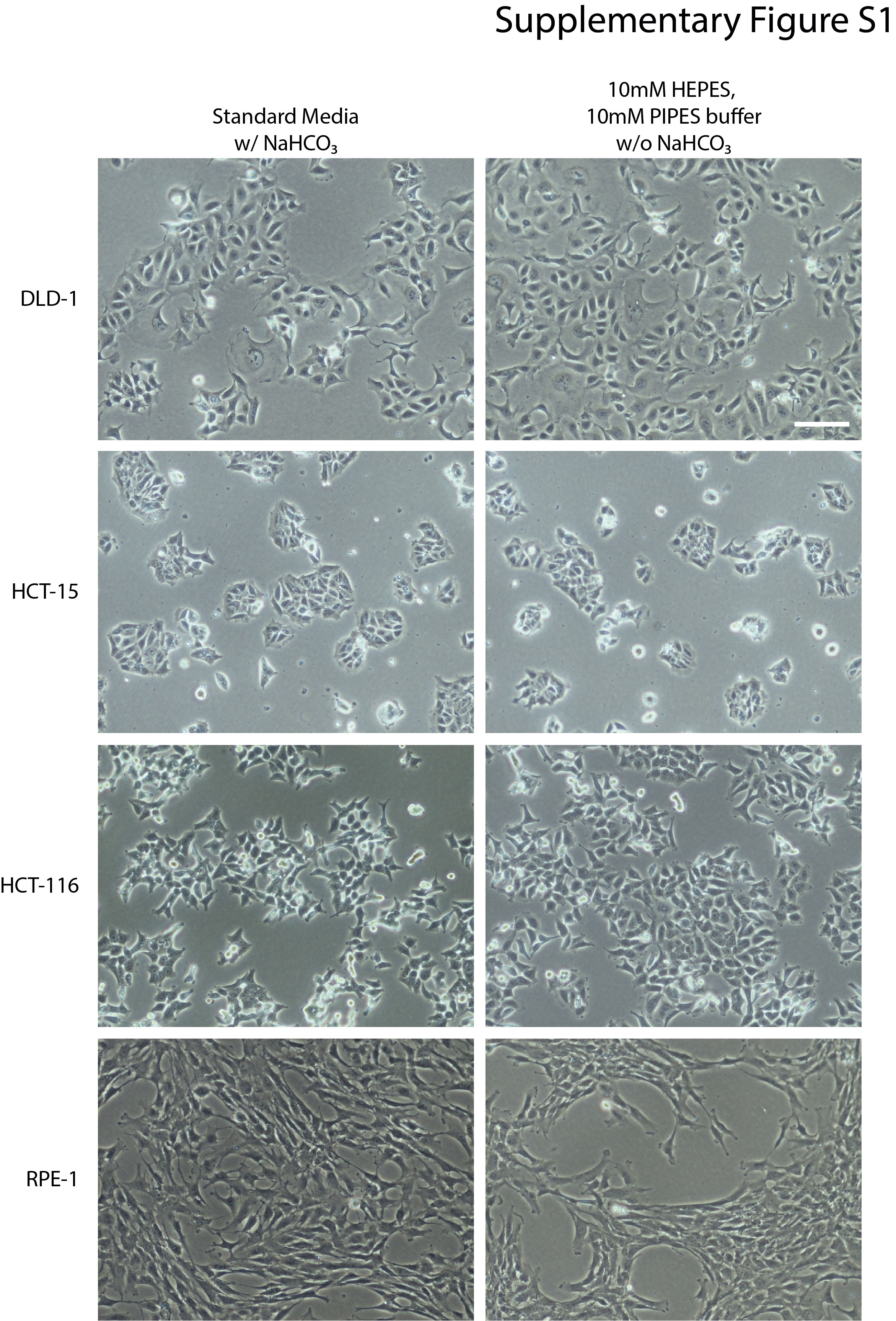


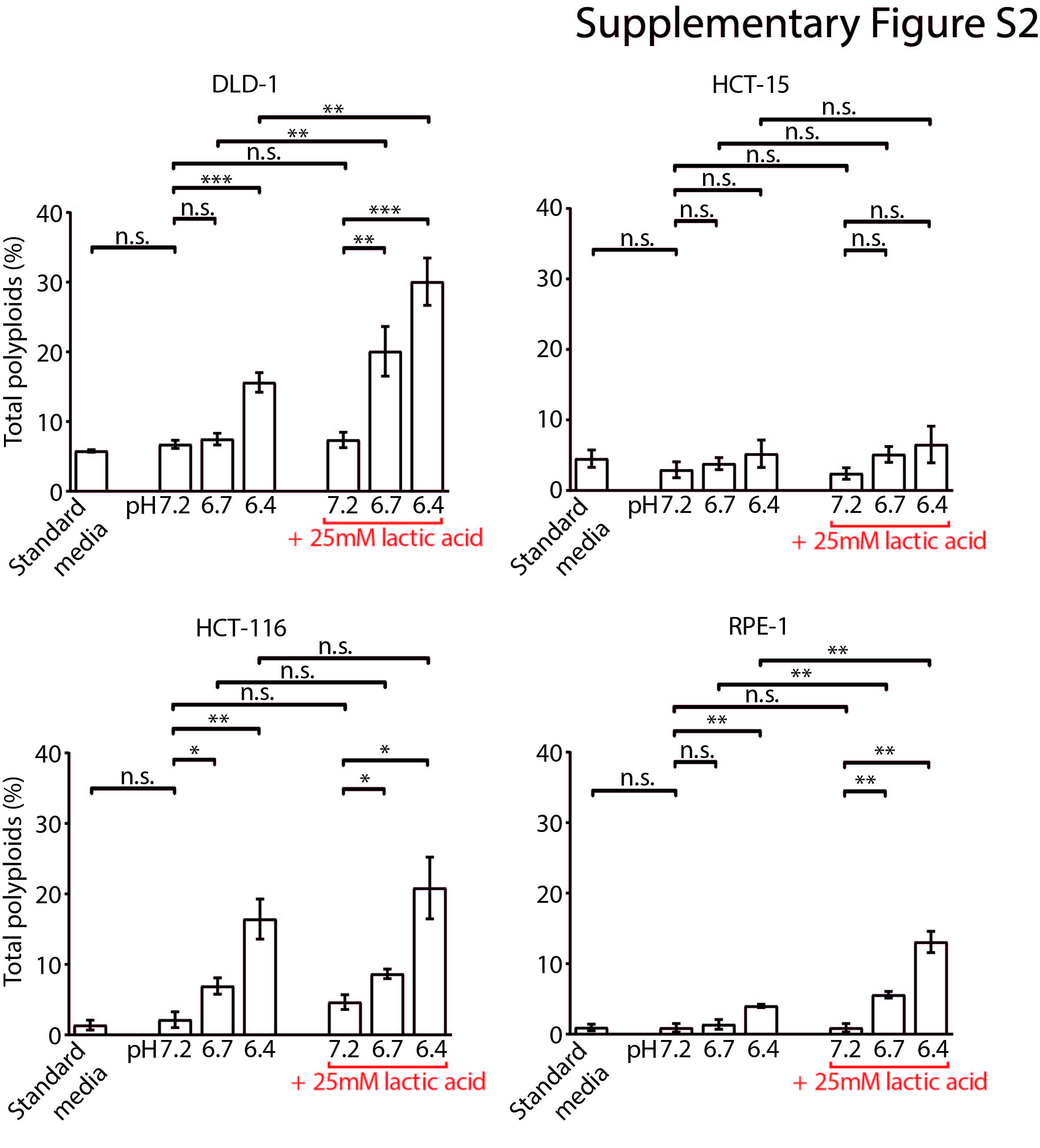


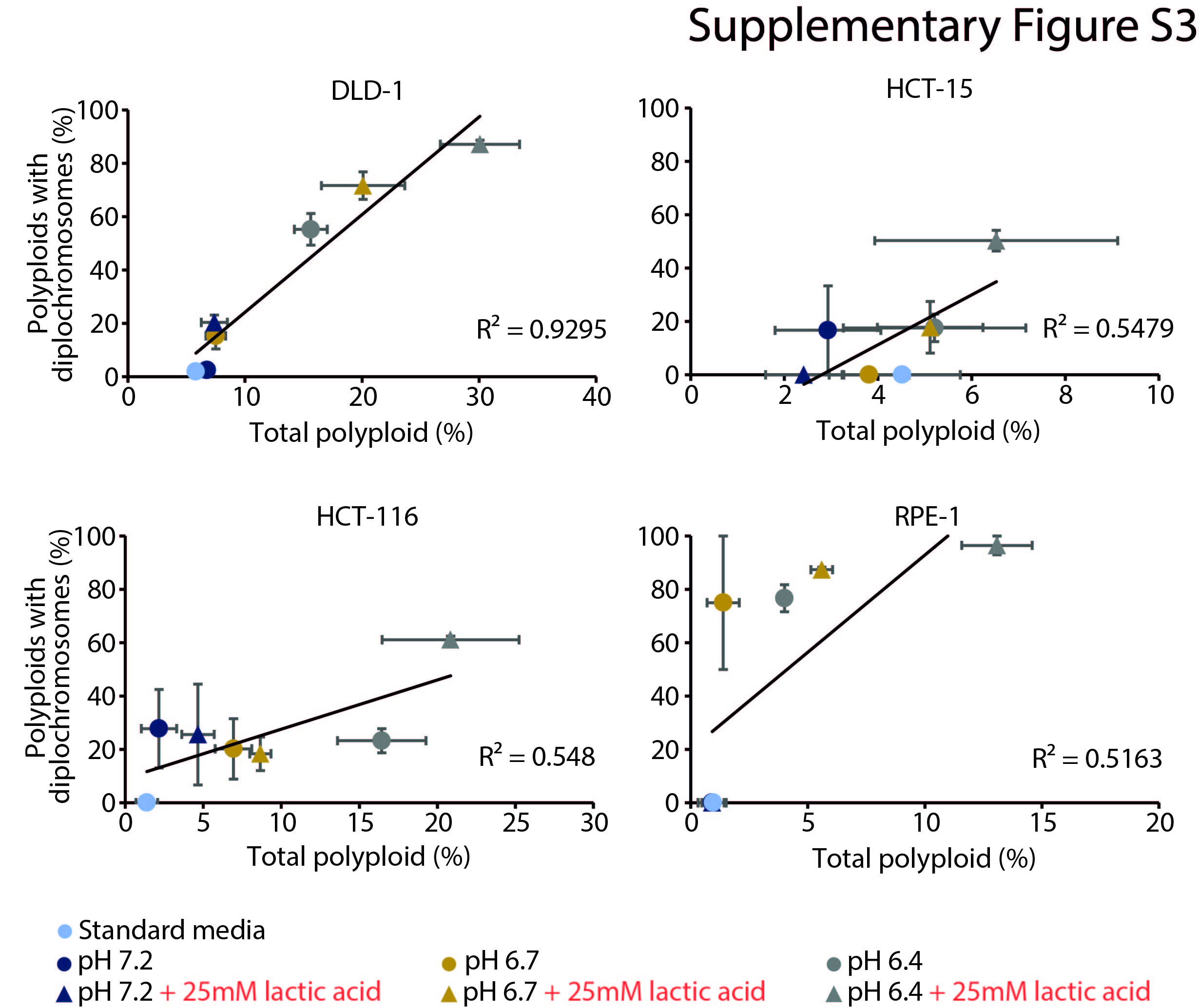


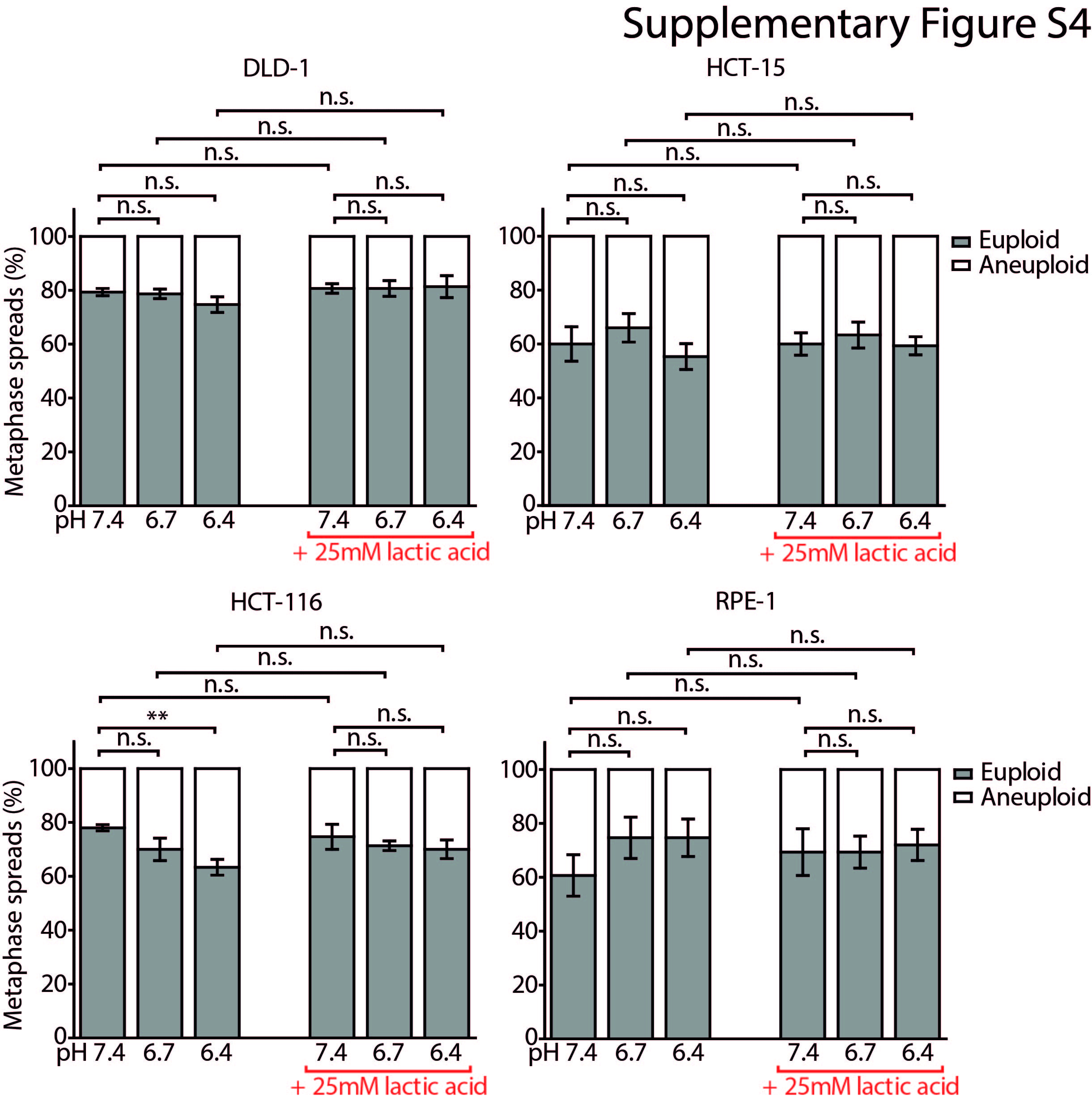

Supplement: Supplementary file 1 — Supplementary information [file 41598_2018_20186_MOESM1_ESM.doc]
